# Infant HIV transmission despite maternal viral suppression: a case of post-weaning seroconversion

**DOI:** 10.1093/jac/dkag072

**Published:** 2026-03-04

**Authors:** Shadia Nakalema, Diana Namuddu, Isabella Kyohairwe, Irene Nakatudde, Thokozile Malaba, Landon Myer, Angela Colbers, Helen Reynolds, Jim Read, Mohammed Lamorde, Saye Khoo, Catriona Waitt

**Affiliations:** Infectious Diseases Institute, College of Health Sciences, Makerere University, Kampala, Uganda; Faculty of Health and Life Sciences, University of Liverpool, Liverpool, UK; Infectious Diseases Institute, College of Health Sciences, Makerere University, Kampala, Uganda; Infectious Diseases Institute, College of Health Sciences, Makerere University, Kampala, Uganda; Infectious Diseases Institute, College of Health Sciences, Makerere University, Kampala, Uganda; School of Public Health, Division of Epidemiology and Biostatistics, University of Cape Town, Cape Town, South Africa; School of Public Health, Division of Epidemiology and Biostatistics, University of Cape Town, Cape Town, South Africa; Radboud Institute of Health Sciences, Radboud University Medical Centre, Nijmegen, Netherlands; Faculty of Health and Life Sciences, University of Liverpool, Liverpool, UK; Centre for Experimental Therapeutics, University of Liverpool, Liverpool, UK; Clinical Sciences Department, Global Health Trials Unit, Liverpool School of Tropical Medicine, Liverpool, UK; Infectious Diseases Institute, College of Health Sciences, Makerere University, Kampala, Uganda; Faculty of Health and Life Sciences, University of Liverpool, Liverpool, UK; Centre for Experimental Therapeutics, University of Liverpool, Liverpool, UK; Infectious Diseases Institute, College of Health Sciences, Makerere University, Kampala, Uganda; Faculty of Health and Life Sciences, University of Liverpool, Liverpool, UK; Department of Women’s and Children’s Health, University of Liverpool, Liverpool, UK

To the Editor-in-Chief,

Despite widespread availability of antiretroviral therapy (ART) and comprehensive prevention of vertical transmission (PVT) programmes, rare cases of postnatal HIV transmission continue to occur. Maternal viral suppression during pregnancy and breastfeeding has dramatically reduced transmission risk, with global guidelines now recommending maternal ART as the cornerstone of prevention.^1^ Current evidence estimates the overall risk of pregnancy and postnatal HIV transmission to be less than 1% in mothers who are fully suppressed on ART.^2^ However, we report a case that illustrates a complex diagnostic and clinical challenge: infant HIV seroconversion following cessation of breastfeeding, despite sustained maternal virological suppression and adherence to all PVT interventions.

The mother was a 19-year-old primigravida in Kampala, Uganda, diagnosed with HIV at 33 weeks gestation through routine antenatal screening. She was enrolled in the DolPHIN-2 study, a randomized controlled trial comparing dolutegravir-based versus efavirenz-based ART for late-presenting pregnant women (NCT03249181).^3^ On the day of diagnosis, she initiated standard ART consisting of tenofovir disoproxil fumarate, lamivudine and efavirenz (TDF/3TC/EFV). HIV-1 RNA was 106 539 copies/mL at baseline. Within 3 months of treatment initiation, she achieved plasma viral suppression (<50 copies/mL) and maintained this throughout the follow-up period (Figure 1).

**Figure 1.**
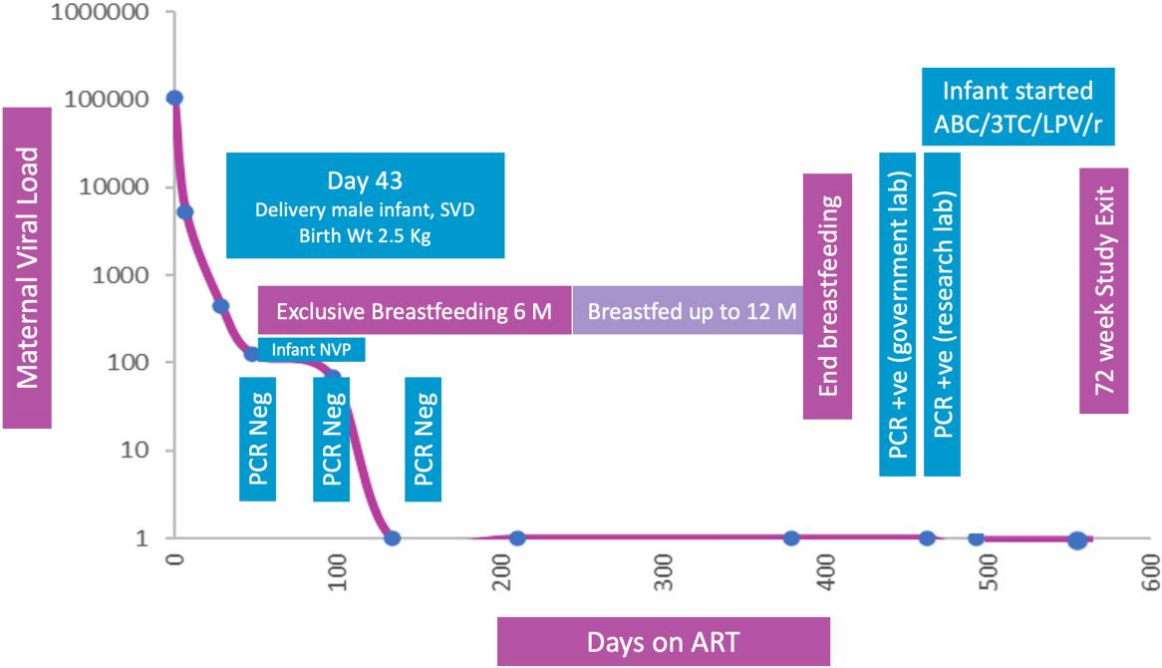
Maternal HIV viral load and infant PCR timeline across the 72-week follow-up period of the DolPHIN2 study.

The infant was delivered vaginally at term, weighing 3.1 kg. The birth HIV PCR was negative. In accordance with national and WHO guidelines,^1^ the infant received 6 weeks of nevirapine prophylaxis and was exclusively breastfed for the first 6 months. Complementary feeding was introduced thereafter, and breastfeeding continued until 12 months of age. The infant underwent serial HIV testing at birth, 6 weeks, 3 months, 6 months, 9 months and 11 months, with all results negative. At 14-months of age, approximately 6 weeks after cessation of breastfeeding, the infant tested positive for HIV by PCR. Confirmatory viral load was 90 210 copies/mL. Resistance testing was performed and revealed no major mutations. However, sequencing identified multiple polymorphisms shared between the mother and infant, suggesting linked transmission. No additional HIV exposure risks (e.g. wet nursing, transfusion or sexual abuse) were identified. The child was initiated on ART and referred to a paediatric HIV clinic for continued care and follow-up.

This case highlights the residual risk of HIV transmission through breastfeeding, even when maternal viral suppression is achieved and PVT guidelines are followed. Pharmacologically, antiretroviral exposure via both placental transfer and breastmilk can result in low-level, subtherapeutic drug concentrations in infants, which may transiently suppress viral replication and influence early HIV diagnostics. Efavirenz demonstrates moderate placental and breastmilk penetration, with a cord-to-maternal plasma (C:M) ratio of 0.49–0.81 and milk-to-plasma (M:P) ratio of 0.54–1.23, producing infant plasma concentrations in the range of ∼100–300 µg/L, detectable but well below therapeutic targets.^4^ Lamivudine shows relatively higher transfer, with a C:M ratio of 0.93–1.22 and M:P ratio of 0.55–3.34, resulting in measurable systemic exposure in the infant.^4^ Tenofovir transfers poorly across the placenta (C:M ratio 0.59–1.20) and minimally into breast milk (M:P 0.015–0.07), contributing little to infant drug levels.^4^

In this case, the timing of HIV acquisition remains uncertain. If infection occurred *in utero*, placental transfer of maternal ART could have partially suppressed viral replication before birth. Postnatal nevirapine prophylaxis, administered orally for 6 weeks, would have added further systemic antiviral pressure. Subsequently, ongoing exposure to maternal ART through breastmilk could have contributed additional low-level antiviral activity. The cumulative effect of *in utero* ART exposure, early nevirapine prophylaxis and breastmilk-mediated drug transfer could theoretically suppress plasma HIV RNA below the analytical sensitivity of standard nucleic acid amplification tests (∼20–50 copies/mL), producing transient false-negative PCR results during prophylaxis and breastfeeding.^5^

This pharmacokinetic and pharmacodynamic interplay plausibly explains the observed pattern of serial negative PCR tests throughout breastfeeding, with detectable viraemia only after drug exposure declined post-weaning, consistent with findings from South African cohorts.^6,7^

Although intrapartum or early postpartum transmission cannot be fully excluded, given the mother had not achieved viral suppression at delivery after presenting with a baseline viral load above 100 000 copies/mL, the infant’s pattern of repeated negative PCRs during breastfeeding, followed by a positive test 6 weeks post-weaning, more strongly suggests delayed detection or post-weaning transmission. HIV can be transmitted antenatally, intrapartum or through breastfeeding, and breastmilk itself contains both cell-free and cell-associated HIV; the latter can persist despite maternal antiretroviral therapy and is believed to play a key role in postnatal transmission.^8,9^ In this context, pharmacological suppression from placental transfer, infant nevirapine prophylaxis and ongoing low-level drug exposure through breastmilk may have reduced viral replication below assay detection thresholds. Resistance testing identified no major mutations to integrase strand transfer inhibitors or protease inhibitors in either mother or infant, but multiple shared polymorphisms supported a linked infection. The absence of major resistance likely reflects the limited selective pressure created by subtherapeutic antiretroviral exposure, which can dampen replication without reliably selecting for resistant variants.

These findings have significant therapeutic and programmatic implications. Whilst standard infant prophylaxis regimens, typically 6 weeks of nevirapine, remain effective in many settings, widespread maternal ART and potential subtherapeutic drug exposure to the infant may transiently suppress viral replication without fully preventing infection. Risk-stratified approaches, including extended or combination prophylaxis for infants born to mothers with high or unsuppressed viral loads, should be considered to further reduce residual transmission. Early infant diagnosis algorithms should incorporate timely nucleic acid testing at birth, 4–6 weeks and post-weaning, with consideration of more sensitive assays capable of detecting cell-associated virus or early viral replication. Post-weaning testing is particularly critical to identify infections that may be masked during prophylaxis or breastfeeding. Integrated maternal-infant care models, including strengthened virological monitoring, adherence support and individualized counselling on feeding practices, are essential to optimize prevention of mother-to-child transmission and ensure timely initiation of antiretroviral therapy for infants who acquire HIV.

Finally, whilst ‘Undetectable = Untransmittable’ (U = U) is well-established for sexual transmission, its application to breastfeeding requires nuance.^10^ Breastfeeding involves prolonged infant mucosal exposure to cell-free and cell-associated virus. Rare postnatal transmissions have occurred despite sustained maternal viral suppression,^6,7^ indicating that U = U messaging cannot be directly extrapolated to breastfeeding. Evidence-based communication should support mothers on effective ART in their feeding choices whilst clearly conveying residual, albeit low, transmission risk, thereby maintaining accuracy and sensitivity in public health messaging. Overall, this case emphasizes the need to reassess early infant diagnostic strategies, consider the pharmacologic effects of maternal and infant ART exposure and continue research into post-weaning surveillance and breastmilk virology to optimize clinical guidance and programmatic policies.

## Data Availability

As this is a case report, all relevant data are reported in the text and figure.
